# Comparative Effects of Femoral Nerve Neurodynamic Techniques and Static Stretching on Quadriceps Muscle Mechanical Properties

**DOI:** 10.3390/bioengineering13080927

**Published:** 2026-08-15

**Authors:** Pinelopi Anestaki, Dimitris Mandalidis, Eleftherios Paraskevopoulos

**Affiliations:** Laboratory of Sports Physical Therapy, Division of Sport Medicine and Biology of Exercise, School of Physical Education and Sport Science, National and Kapodistrian University of Athens, 17237 Athens, Greecedmndldis@phed.uoa.gr (D.M.)

**Keywords:** neurodynamics, femoral nerve, MyotonPRO, muscle mechanical properties

## Abstract

Background: Neurodynamic techniques are commonly used to improve neural mobility and musculoskeletal function, but their effects on muscle mechanical properties remain unclear. This study investigated the acute effects of femoral nerve slider, femoral nerve tensioner, and static stretching interventions on the rectus femoris and vastus medialis muscles. Methods: Thirty healthy adults (18–35 years) were non-randomly allocated using a predetermined alternating sequence to a femoral nerve slider (n = 10), femoral nerve tensioner (n = 10), or static stretching group (n = 10). No sham or no-intervention control group was included. Muscle mechanical properties were assessed before and immediately after the intervention using the MyotonPRO (Myoton AS, Tallinn, Estonia). Outcomes included muscle tone, stiffness, logarithmic decrement, relaxation time, and creep. A three-way mixed repeated-measures ANOVA was performed. Results: No statistically significant differences in pre–post changes were detected among the three intervention groups for any of the examined mechanical parameters. Significant main effects of time were found for stiffness (*p* = 0.026) and creep (*p* = 0.005), indicating overall reductions from pre- to post-assessment. Significant time × muscle interactions were identified for relaxation time (*p* = 0.029) and creep (*p* = 0.002), indicating different temporal patterns between the rectus femoris and vastus medialis. Following Holm adjustment of the follow-up comparisons, the pre–post reduction in the vastus medialis remained statistically significant for creep but not for relaxation time. The rectus femoris demonstrated greater stiffness and logarithmic decrement values, whereas the vastus medialis exhibited greater relaxation time values. Conclusions: No statistically significant differences in immediate pre–post changes in quadriceps muscle mechanical properties were detected among the femoral nerve slider, femoral nerve tensioner, and static stretching groups. Temporal and muscle-specific patterns were observed for selected mechanical parameters; however, these findings should not be interpreted as evidence of equivalence between the interventions.

## 1. Introduction

Neurodynamic techniques are commonly used in musculoskeletal rehabilitation to restore the physiological mobility and mechanical function of the peripheral nervous system [1]. These interventions aim to improve neural excursion, reduce mechanosensitivity, optimize intraneural fluid dispersion, and facilitate normal interactions between neural and surrounding musculoskeletal tissues [2].

Among neurodynamic interventions, slider and tensioner techniques represent two distinct approaches. Slider techniques involve simultaneous movements that increase neural tension at one joint while reducing it at another, promoting nerve excursion with relatively lower strain [3]. In contrast, tensioner techniques elongate the neural bed at multiple joints simultaneously, resulting in greater neural strain and mechanical loading [3]. Previous biomechanical investigations have demonstrated that slider techniques produce larger nerve excursion with less tensile stress, whereas tensioner techniques induce greater strain within neural tissues [3,4,5]. Despite these biomechanical differences, both techniques are widely applied in clinical practice for the management of musculoskeletal and neuromuscular disorders.

Recent evidence suggests that the effects of neurodynamic interventions extend beyond purely mechanical neural mobilization [2]. Experimental and clinical studies indicate that neural mobilization may influence neurophysiological, neuroimmune, and neuromuscular mechanisms associated with pain modulation and tissue homeostasis [2,6,7,8,9]. Additionally, tension-based neurodynamic interventions have been associated with changes in muscle function, locomotion, mechanosensitivity, and peripheral nerve biomechanics [2]. Since muscle mechanical properties may be influenced by interventions targeting peripheral nerves, alterations in neural mechanics may acutely modulate the mechanical behaviour of skeletal muscle tissue.

Mechanical muscle properties such as tone, stiffness, elasticity, and viscoelastic behavior are increasingly recognized as clinically relevant indicators of neuromuscular function and tissue condition [10]. Evidence supporting the validity of MyotonPRO-derived stiffness measurements includes significant associations with shear-wave ultrasound elastography-derived measures of tissue stiffness [11]. Myotonometric parameters include oscillation frequency, representing muscle tone; dynamic stiffness, reflecting resistance to external deformation; logarithmic decrement, associated with tissue elasticity; and relaxation time and creep, which describe viscoelastic properties of the muscle [12] (Table 1). Objective assessment of these parameters may provide valuable insight into acute neuromechanical adaptations following neurodynamic interventions [13].

The quadriceps muscle group plays an important role in lower-limb function, force generation, and dynamic knee stability during athletic and functional activities [14,15]. In particular, the rectus femoris and vastus medialis muscles are primarily engaged during movements requiring upper-leg force production, such as sprinting, jumping, and executing powerful shots on goal [15]. Alterations in quadriceps mechanical properties may influence movement efficiency and neuromuscular performance [16,17]. Although previous studies have investigated the effects of neurodynamic techniques on pain [18,19,20,21], range of motion [22], flexibility [22], and autonomic responses [9], limited evidence exists regarding their acute effects on objectively measured muscle mechanical properties. Furthermore, comparative evidence examining femoral nerve slider and tensioner techniques on quadriceps muscle mechanical behavior remains scarce.

Therefore, the aim of this study was to investigate the acute effects of femoral nerve slider, femoral nerve tensioner, and static stretching interventions on the mechanical properties of the rectus femoris and vastus medialis muscles using the MyotonPRO. It was hypothesized that neurodynamic interventions would induce significant acute alterations in quadriceps mechanical properties and that tensioner techniques would produce greater changes compared with slider techniques and static stretching.

## 2. Materials and Methods

This study employed a prospective, non-randomized controlled experimental design to investigate the acute effects of femoral nerve slider, femoral nerve tensioner, and static stretching interventions on the mechanical properties of the rectus femoris and vastus medialis muscles. Participants were allocated into three intervention groups (femoral nerve slider, femoral nerve tensioner, and static stretching) using a predetermined alternating allocation sequence. Because this procedure was not random, allocation concealment was not implemented.

The study protocol was prospectively registered prior to participant recruitment (TCTR20260225006) and was approved by the Ethics Committee of the Department of Physical Education and Sports Science, National and Kapodistrian University of Athens (Approval Protocol Number: 1952/18-03-2026). The study was conducted in accordance with the principles of the Declaration of Helsinki for research involving human participants. Prior to participation, all individuals received verbal and written information regarding the study procedures and provided written informed consent.

### 2.1. Sample Size Calculation

An a priori sample size calculation was performed using GPower software (Version 3.1.9.7, Heinrich-Heine-University Düsseldorf, Germany), using the F tests, repeated-measures ANOVA, and the within–between interaction module. In accordance with the prospective trial registration, muscle stiffness, tone, and elasticity were designated as primary outcomes, whereas relaxation time and creep were designated as secondary outcomes. In the absence of directly comparable studies examining the acute effects of femoral nerve neurodynamic interventions on MyotonPRO-derived muscle mechanical properties, a moderate effect size (f = 0.25) was assumed according to Cohen’s conventional classification for ANOVA-based analyses. This assumption was considered appropriate given the previously reported acute effects of neurodynamic and neuromuscular interventions on mechanical and functional outcomes [23]. Assuming α = 0.05, statistical power of 80%, three intervention groups, four repeated observations, a correlation among repeated measures of 0.50, and a nonsphericity correction of ε = 1.0, a total sample size of 30 participants was required (numerator df = 6, denominator df = 81; actual power = 0.809). The four repeated observations represented measurements obtained from two muscles at two assessment time points (pre and post). The GPower calculation was based on the overall within–between interaction and did not separately model time and muscle as distinct within-subject factors. To further characterize the detectable intervention-specific temporal effect with the achieved sample, a sensitivity analysis was performed using three intervention groups and two assessment time points. With N = 30, α = 0.05, power = 0.80, a correlation among repeated measures of 0.50, and ε = 1.0, the minimum detectable Time × Intervention effect was f = 0.300. Thus, smaller intervention-specific temporal effects may not have been detectable.

### 2.2. Participants

Healthy adults of both sexes, aged between 18 and 35 years, voluntarily participated in the study. Participants were recruited consecutively throughout the study period and allocated to one of three intervention groups (femoral nerve slider, femoral nerve tensioner, and static stretching) using a predetermined alternating allocation sequence at the time of enrollment to ensure relatively balanced group sizes. Due to the nature of the allocation procedure, allocation concealment was not implemented.

Inclusion criteria consisted of the absence of lower-limb musculoskeletal injury during the previous six months, absence of pain or functional limitation during the movements required by the protocol, and general good health status. Exclusion criteria included a history or presence of neurological or neuromuscular disorders, active musculoskeletal injury or recent lower-limb surgery, and any condition that could affect neuromuscular function or increase the risk of adverse responses during the interventions. Due to the nature of the interventions, participant and therapist blinding was not feasible. However, all measurements and interventions were performed by the same investigator using standardized procedures in order to minimize measurement bias.

### 2.3. Physical Activity Assessment

Physical activity levels were assessed using the Greek version of the International Physical Activity Questionnaire–Short Form (IPAQ-SF) after obtaining permission to use the questionnaire from its developers [24]. The IPAQ-SF was administered on the same day as the MyotonPRO assessment and assessed participants’ physical activity during the preceding seven days. Participants were classified into low-, moderate-, or high-physical-activity categories according to the standardized IPAQ scoring protocol [25]. Physical activity data were collected to describe the sample and examine baseline comparability between intervention groups. The IPAQ-SF was used solely to characterize the physical activity profile of the study sample.

### 2.4. Experimental Procedures

Before data collection, all participants received detailed information regarding the purpose and procedures of the study. A brief familiarization session with the testing procedures and equipment was conducted to ensure participant understanding and comfort.

To minimize the influence of external factors on muscle mechanical properties, several standardization procedures were applied. All measurements were performed within a similar time period during the day in order to reduce potential circadian influences. Participants were instructed to refrain from strenuous lower-limb exercise for at least 24 h before testing and to avoid excessive consumption of stimulants such as caffeine on the day of assessment. Additionally, all measurements and interventions were conducted under standardized environmental and testing conditions.

Initially, baseline measurements of the mechanical properties of the quadriceps muscles were obtained using the MyotonPRO device. Assessments were performed on the dominant leg on predetermined anatomical locations of the rectus femoris and vastus medialis muscles while participants remained relaxed in a supine position. Lower-limb dominance was determined based on the participant’s preferred leg for kicking a ball [26].

Following baseline assessment, participants underwent the intervention corresponding to their allocated group, consisting of either a femoral nerve slider technique, femoral nerve tensioner technique, or static quadriceps stretching. All interventions were performed within a pain-free and safe range of motion without provoking discomfort or symptoms of neural irritation. Immediately after completion of the intervention, post-intervention measurements were obtained using the same assessment procedures and equipment in order to evaluate acute changes in muscle mechanical properties.

All interventions were administered under the direct supervision of the investigator to ensure procedural standardization and consistency in execution. Throughout the interventions, participants were instructed to maintain a calm and controlled breathing pattern to minimize the potential influence of respiration on muscle relaxation and neuromuscular activity [27]. No adverse events or interruptions of the intervention procedures were reported.

### 2.5. Intervention Procedures

#### 2.5.1. Femoral Nerve Slider Technique

**Figure 1:**
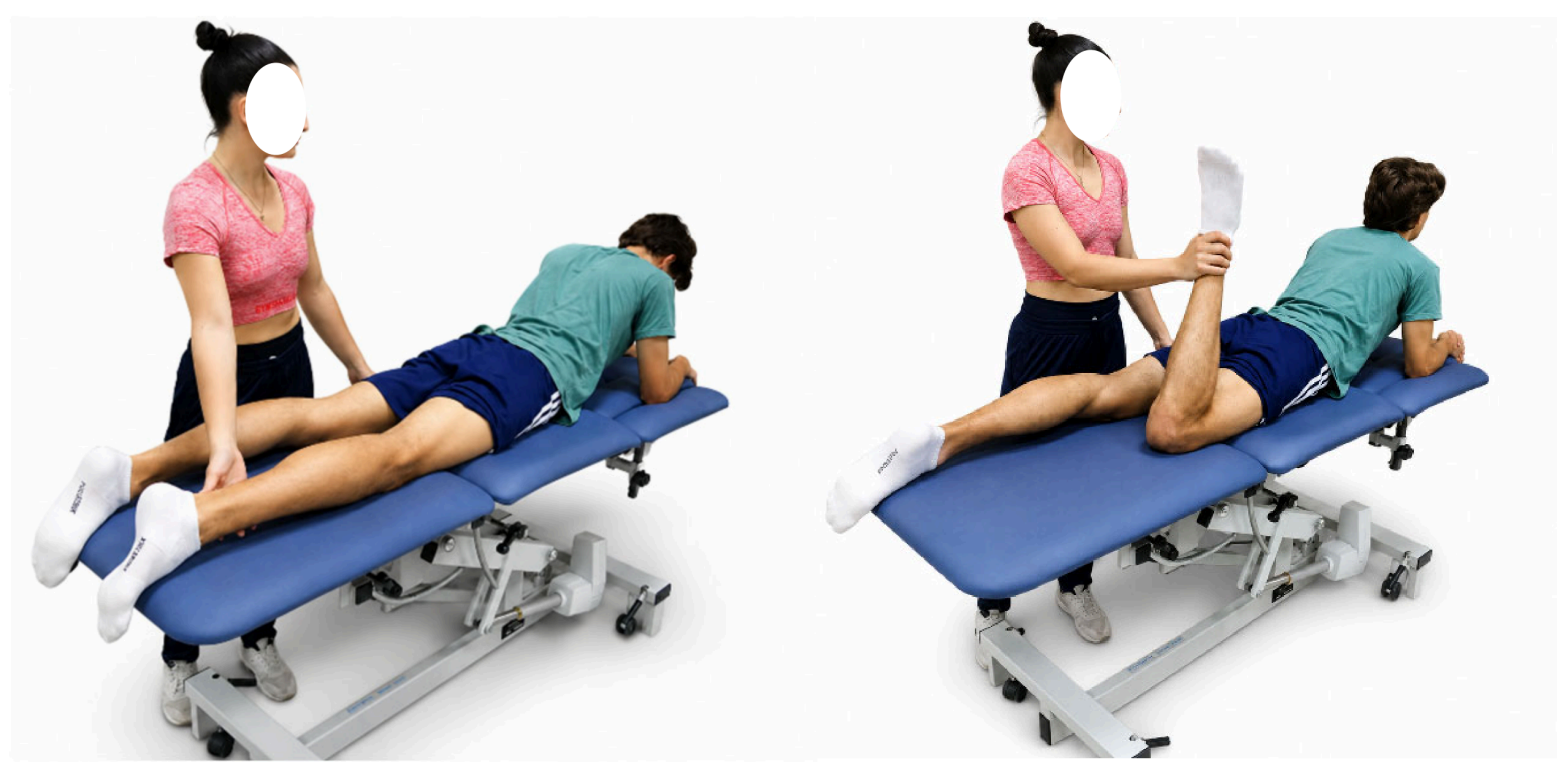
Femoral nerve slider neurodynamic technique performed in the prone position. The figure demonstrates the initial (**left**) and final (**right**) positions of the slider technique.

Participants assigned to the femoral nerve slider group underwent a neurodynamic slider technique targeting the femoral nerve in the prone-lying position (Figure 1). The hip was maintained in a neutral position throughout the procedure, without deliberate hip extension, while the pelvis and lumbar spine were maintained in a neutral, relaxed position without compensatory movement. In the initial position, the cervical spine was flexed and the knee was extended. Participants then actively performed knee flexion simultaneously with cervical extension, followed by the reverse movement consisting of knee extension combined with cervical flexion. Thus, movement occurred primarily at the knee and cervical spine, while the hip, pelvis, and lumbar spine remained in their prescribed positions throughout the procedure. This alternating movement sequence was intended to increase mechanical loading at one end of the neural bed while simultaneously reducing it at the other, thereby promoting neural excursion while limiting sustained neural strain. The movements were performed actively by the participants under the supervision of the investigator and through each participant’s available symptom-free range of motion rather than to a predetermined joint angle. The investigator monitored movement amplitude, participant positioning, and technique throughout the procedure and provided verbal correction when necessary to maintain the prescribed movement sequence and symptom-free range. The technique was performed rhythmically at a cadence of 40 beats per minute (bpm), standardized using a metronome [28,29]. Each movement phase corresponded to one metronome beat (approximately 1.5 s); thus, knee flexion combined with cervical extension was performed on one beat, followed by knee extension combined with cervical flexion on the subsequent beat. Accordingly, one complete movement cycle comprised two beats and lasted approximately 3 s. The procedure was performed continuously for 180 s, corresponding to approximately 60 complete movement cycles. Neural loading was individually limited according to each participant’s symptom response. Specifically, movements were required to remain pain-free and not provoke excessive stretching sensations, paresthesia, or other symptoms suggestive of neural irritation. If any such symptoms occurred, the movement amplitude was reduced until the procedure could again be performed comfortably within a symptom-free range. Thus, the slider procedure was standardized with respect to participant positioning, movement sequence, cadence, intervention duration, and approximate number of movement cycles, whereas movement amplitude was individualized according to each participant’s symptom-free range. Joint angles and perceived stretch or discomfort intensity were not quantitatively recorded; therefore, identical mechanical loading across participants cannot be assumed. A video demonstrating the complete movement sequence is provided in Supplementary Video S1.

#### 2.5.2. Femoral Nerve Tensioner Technique

**Figure 2:**
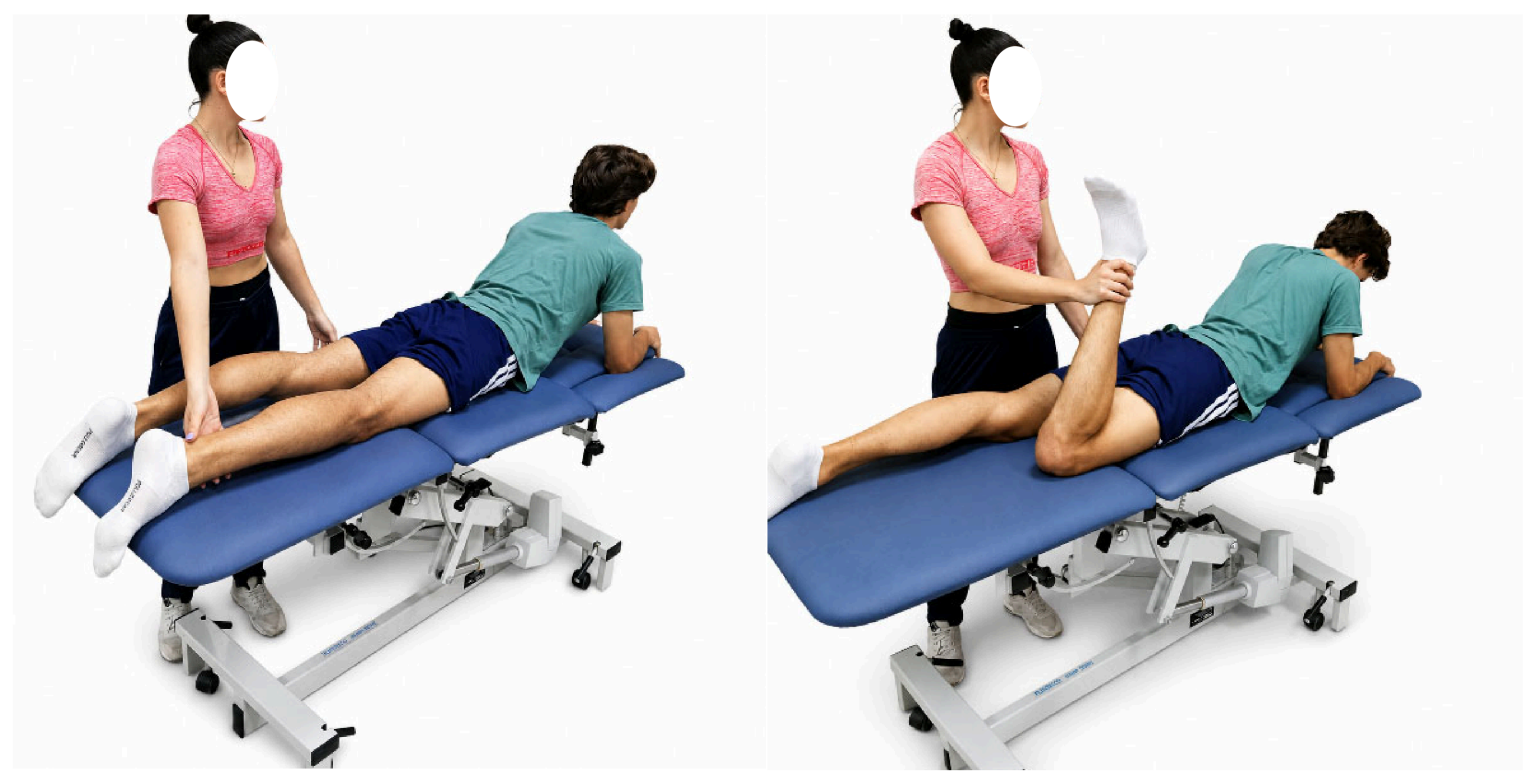
Femoral nerve tensioner neurodynamic technique performed in the prone position. The figure demonstrates the initial (**left**) and final (**right**) positions of the tensioner technique.

Participants assigned to the femoral nerve tensioner group underwent a neurodynamic tensioner technique targeting the femoral nerve in the prone-lying position (Figure 2). The hip was maintained in a neutral position throughout the procedure, without deliberate hip extension, while the pelvis and lumbar spine were maintained in a neutral, relaxed position without compensatory movement. In the initial position, the cervical spine was extended and the knee was extended. Participants then actively performed knee flexion simultaneously with cervical flexion, thereby increasing mechanical loading along the neural bed, followed by the reverse movement consisting of knee extension combined with cervical extension. Thus, movement occurred primarily at the knee and cervical spine, while the hip, pelvis, and lumbar spine remained in their prescribed positions throughout the procedure. In contrast to the slider technique, these coordinated movements were intended to simultaneously increase or decrease mechanical loading at both ends of the neural bed, thereby producing greater neural loading during the tensioning phase. The movements were performed actively by the participants under the supervision of the investigator and through each participant’s available symptom-free range of motion rather than to a predetermined joint angle. The investigator monitored movement amplitude, participant positioning, and technique throughout the procedure and provided verbal correction when necessary to maintain the prescribed movement sequence and symptom-free range. The technique was performed rhythmically at a cadence of 40 beats per minute (bpm), standardized using a metronome [28,29]. Each movement phase corresponded to one metronome beat (approximately 1.5 s); thus, knee flexion combined with cervical flexion was performed on one beat, followed by knee extension combined with cervical extension on the subsequent beat. Accordingly, one complete movement cycle comprised two beats and lasted approximately 3 s. The procedure was performed continuously for 180 s, corresponding to approximately 60 complete movement cycles. Neural loading was individually limited according to each participant’s symptom response. Specifically, movements were required to remain pain-free and not provoke excessive stretching sensations, paresthesia, or other symptoms suggestive of neural irritation. If any such symptoms occurred, the movement amplitude was reduced until the procedure could again be performed comfortably within a symptom-free range. Thus, the tensioner procedure was standardized with respect to participant positioning, movement sequence, cadence, intervention duration, and approximate number of movement cycles, whereas movement amplitude was individualized according to each participant’s symptom-free range. Joint angles and perceived stretch or discomfort intensity were not quantitatively recorded; therefore, identical mechanical loading across participants cannot be assumed. A video demonstrating the complete movement sequence is provided in Supplementary Video S2.

#### 2.5.3. Static Stretching

**Figure 3:**
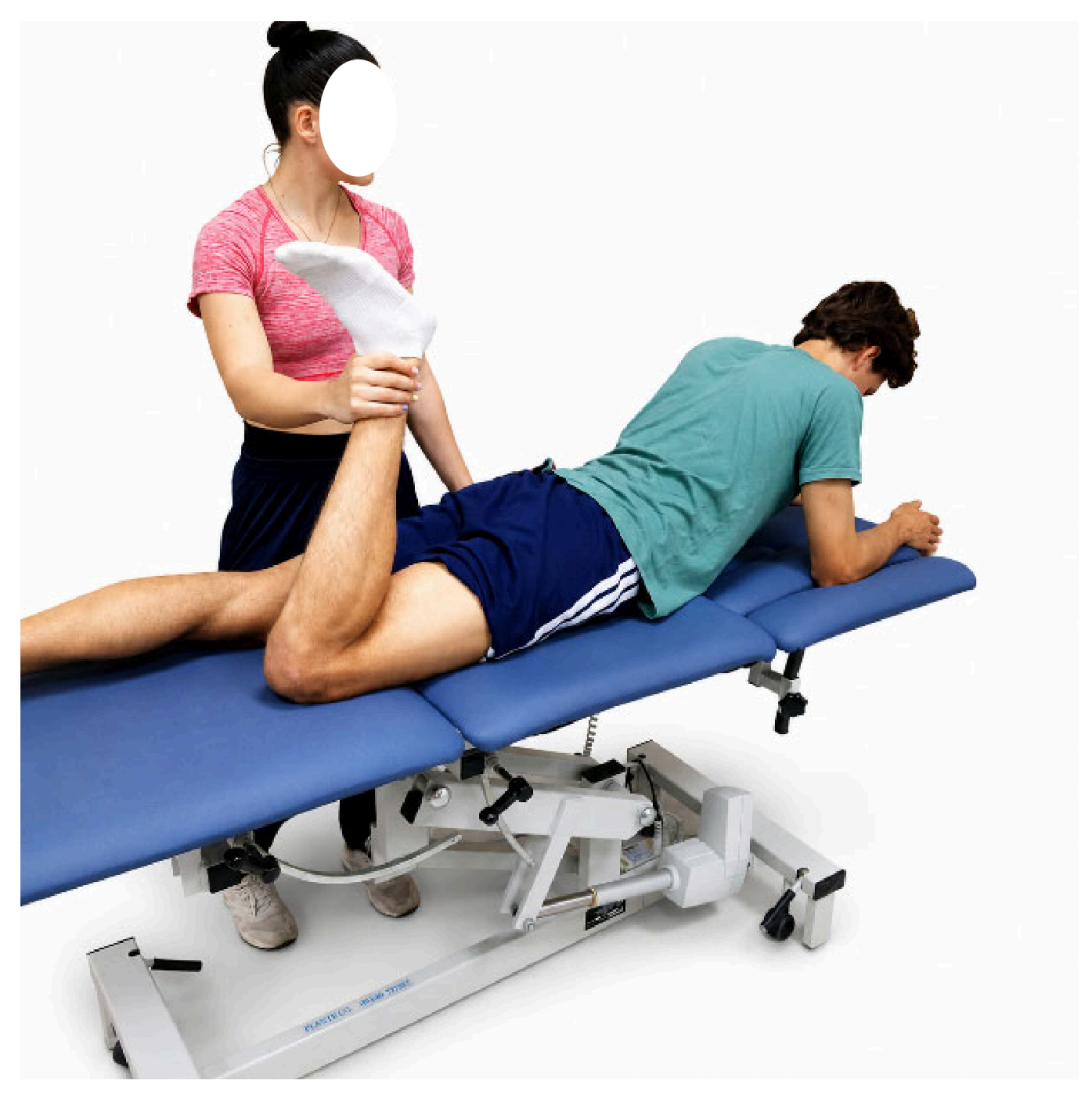
Static stretching technique performed in prone position.

Participants assigned to the static stretching group received a structured passive static stretching intervention targeting the quadriceps muscle group in the prone-lying position (Figure 3). The hip was maintained in a neutral position throughout the procedure, without deliberate hip extension, while the pelvis and lumbar spine were maintained in a neutral, relaxed position without compensatory movement. The cervical spine remained in a comfortable neutral position and was not used as a component of the stretching procedure. The stretch was performed passively, with knee movement manually controlled by the same investigator who administered the neurodynamic interventions. With the participant instructed to remain relaxed, the investigator progressively flexed the knee through the participant’s available range of motion until a clear but tolerable stretching sensation was reported in the anterior thigh, without pain or other discomfort. No predetermined knee-flexion angle was imposed; instead, the stretching amplitude was individualized according to each participant’s tolerance. The endpoint was defined as the maximum knee-flexion position that produced a noticeable but comfortable quadriceps stretch without pain. The investigator maintained the knee at this position throughout each stretching bout without additional movement and monitored the participant’s response. If pain or other discomfort was reported, the degree of knee flexion was reduced until a comfortable stretching sensation was restored. Thus, the stretching procedure was standardized with respect to participant positioning, application by the same investigator, bout duration, rest intervals, and number of bouts, whereas stretching amplitude was individualized according to participant tolerance. Knee-flexion angle and perceived stretching intensity were not quantitatively recorded; therefore, identical mechanical stretching intensity across participants cannot be assumed. Each stretch was held for 30 s, with a 10-s rest interval between consecutive stretching bouts, during which the knee was returned to a relaxed position. The procedure consisted of six 30-s stretching bouts separated by five 10-s rest intervals, resulting in 180 s of active stretching exposure and a total elapsed protocol duration of 230 s. The 180-s active stretching exposure was selected to correspond to the 180-s active intervention period of the two neurodynamic protocols [28,30,31]. Thus, the interventions were matched for active intervention exposure but not for total elapsed protocol duration.

### 2.6. Myotonometric Assessment

Mechanical properties of the rectus femoris and vastus medialis muscles were assessed using the MyotonPRO device (Myoton AS, Tallinn, Estonia), a handheld non-invasive myotonometer that enables objective quantification of muscle mechanical characteristics. The device records brief mechanical oscillations induced by a short-duration impulse delivered to the skin surface overlying the muscle. The following parameters were analyzed: oscillation frequency (Hz), representing muscle tone; dynamic stiffness (N/m); logarithmic decrement, representing elasticity; mechanical stress relaxation time (ms), characterizing tissue recovery following deformation; and creep (C), a dimensionless viscoelastic parameter calculated as the ratio of mechanical stress relaxation time to deformation time (Deborah number). Previous evidence has demonstrated good-to-excellent reliability of the MyotonPRO for the rectus femoris muscle, with intra-rater ICC values ranging from 0.78 to 0.99 and inter-rater ICC values ranging from 0.82 to 0.97. Similarly, vastus medialis assessments have shown excellent inter-rater reliability, with ICC values ranging from 0.91 to 0.96 [32]. In addition to reliability, evidence supporting the validity of MyotonPRO-derived stiffness measurements has been reported through comparison with shear-wave ultrasound elastography. Feng et al. [11] demonstrated significant associations between MyotonPRO-derived stiffness and elastography-derived Young’s modulus for skeletal muscle and tendon, providing evidence of convergent validity for stiffness assessment.

Muscle stiffness, muscle tone, and elasticity were prospectively registered as primary outcomes, whereas relaxation time, creep, perceived stretch discomfort, and knee flexion range of motion were registered as secondary outcomes. Perceived stretch discomfort and knee flexion range of motion were not ultimately collected because priority was given to completing the MyotonPRO reassessment within the approximately one-minute post-intervention assessment window required to capture the immediate mechanical response. This represents a deviation from the prospectively registered protocol.

Measurement sites were selected according to the previous literature investigating quadriceps muscle mechanical properties [33]. For the rectus femoris, the MyotonPRO probe was positioned at two-thirds of the distance from the anterior superior iliac spine to the superior border of the patella and for the vastus medialis, the probe was positioned at 80% of the distance between the anterior superior iliac spine and the knee joint space anterior to the medial collateral ligament. Following identification of the anatomical measurement sites, each location was marked on the skin using a semi-permanent marker to ensure that the same site was assessed during the post-intervention measurement. Post-intervention measurements commenced immediately following completion of the intervention and were initiated within one minute. The rectus femoris was assessed first, followed by the vastus medialis, and this order was maintained for all participants at both measurement time points. Post-intervention measurements commenced immediately following completion of the intervention, with assessment of the rectus femoris initiated within one minute. Each muscle required approximately 5 s to assess, with the vastus medialis assessed immediately after the rectus femoris.

**Figure 4:**
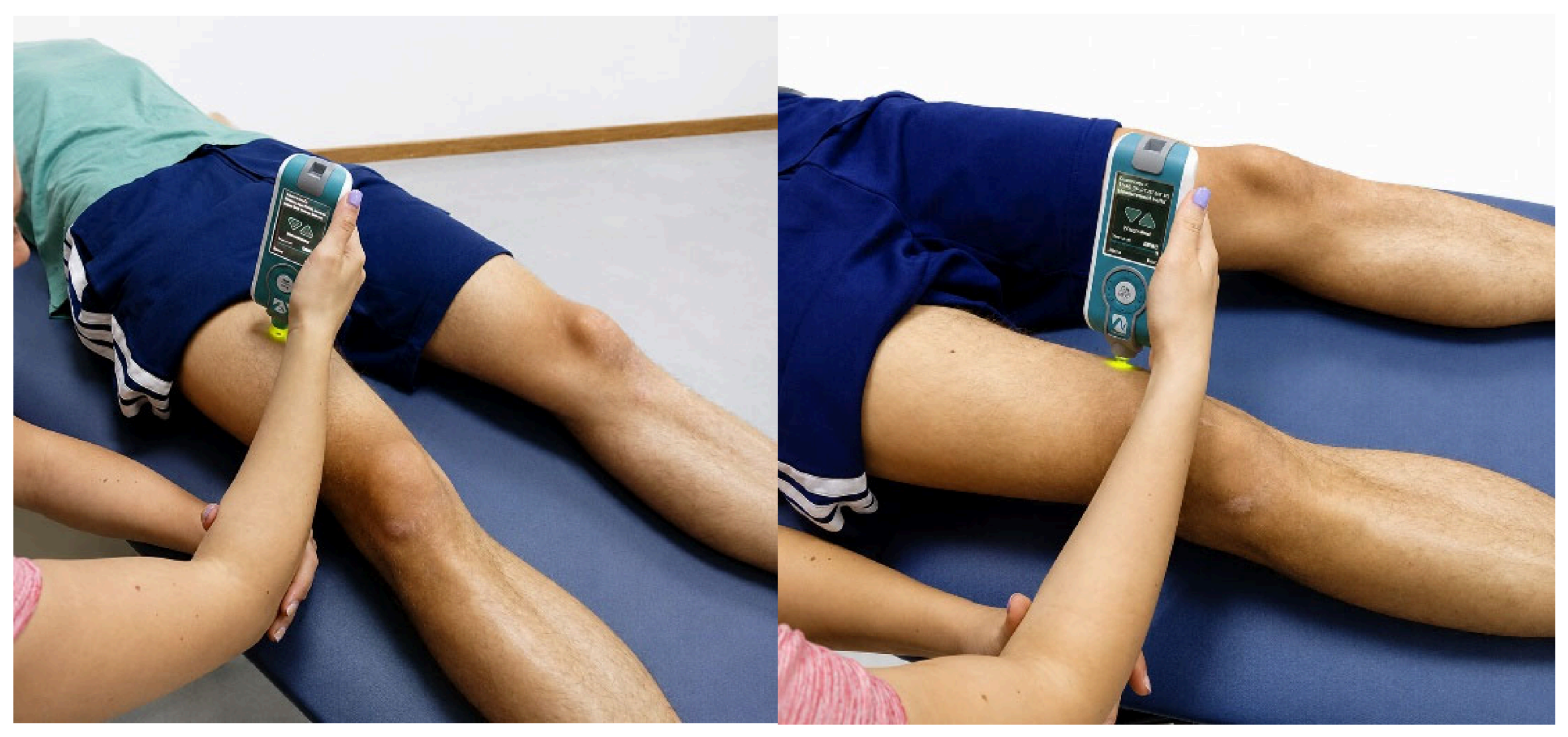
Assessment of rectus femoris (**left**) and vastus medialis (**right**) mechanical properties using the MyotonPRO device.

Measurements were performed with participants in a relaxed and standardized supine position, while the MyotonPRO device was applied perpendicularly to the skin surface (Figure 4). Each measurement sequence consisted of three consecutive scans separated by 15 ms, obtained without removing or repositioning the probe between scans. This procedure minimized potential variability associated with repeated probe placement. Probe application was controlled using the device’s built-in pressure-feedback mechanism; when insufficient or excessive pressure was applied, a red indicator was displayed and measurement acquisition was prevented until appropriate probe pressure was achieved. No measurement sequences required repetition during data collection. The values obtained from the three consecutive scans were averaged and used for statistical analysis. Three consecutive measurements were obtained at each anatomical site, and the mean value was used for statistical analysis.

### 2.7. Statistical Analysis

Statistical analyses were performed using IBM SPSS Statistics software (29.0, IBM Corp., Armonk, NY, USA). Descriptive statistics were calculated for all variables and are presented as mean ± standard deviation (SD). Normality of data distribution was assessed using the Shapiro–Wilk test in combination with visual inspection of Q–Q plots. Homogeneity of variances was evaluated using Levene’s test, while homogeneity of covariance matrices was assessed using Box’s M test. Baseline differences between intervention groups for continuous variables were examined using one-way analysis of variance (ANOVA), whereas sex distribution was compared using Pearson’s chi-square test. Given the small expected cell frequencies for IPAQ-SF classifications, differences among intervention groups in IPAQ-SF categories were assessed using the Fisher–Freeman–Halton exact test.

A three-way mixed repeated-measures analysis of variance (ANOVA) was performed separately for each MyotonPRO parameter. Intervention (slider, tensioner, and static stretching) was entered as the between-subject factor, whereas time (pre-intervention and post-intervention) and muscle (rectus femoris and vastus medialis) were entered as within-subject factors. Main effects and interaction effects, including time × intervention, time × muscle, muscle × intervention, and time × muscle × intervention interactions, were examined. When significant interaction effects were identified, follow-up simple-effect comparisons were performed to further explore the source of the interaction. To account for multiple follow-up comparisons, *p*-values were adjusted using the Holm procedure to control the family-wise error rate. Both unadjusted and Holm-adjusted *p*-values are reported where applicable. Effect sizes were calculated using partial eta squared (η^2^*p*), with values of 0.01, 0.06, and 0.14 interpreted as small, medium, and large effects, respectively. Statistical significance was set at *p* < 0.05.

## 3. Results

**Figure 5:**
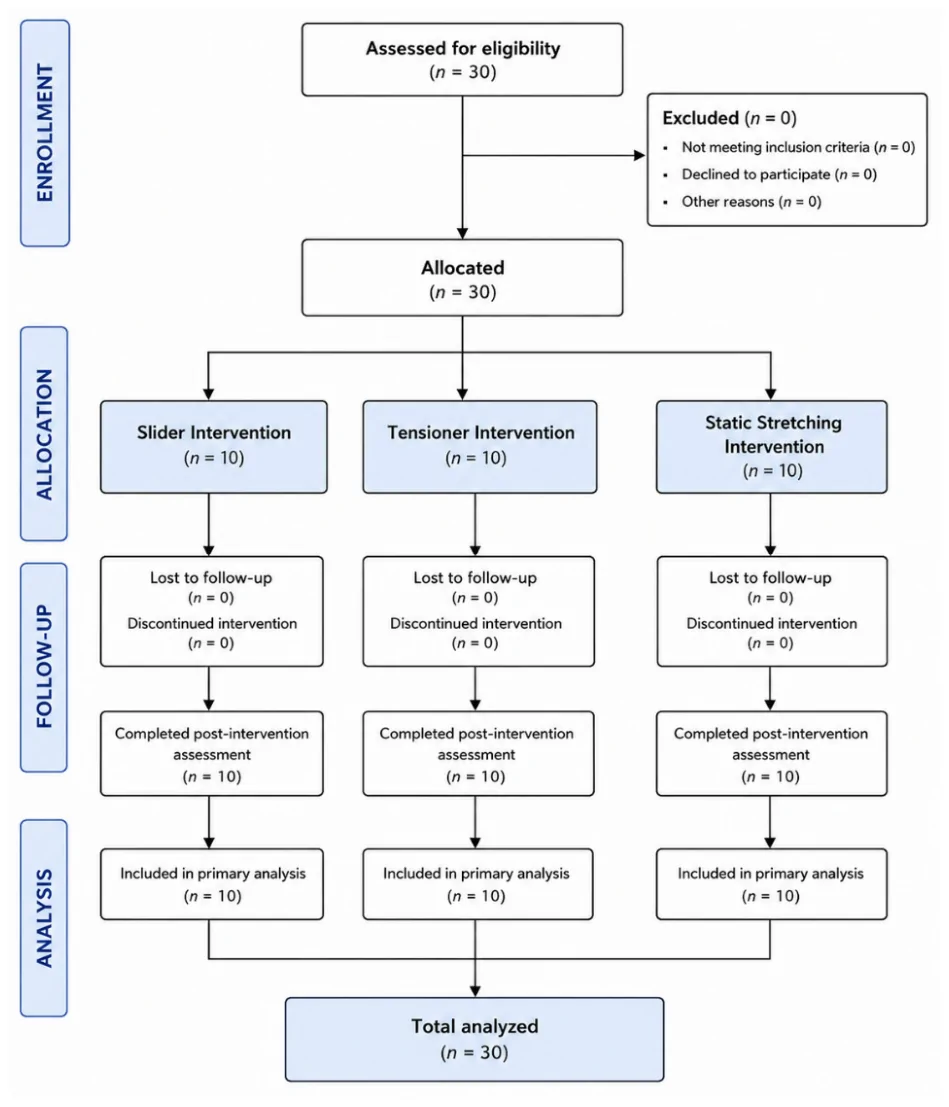
Participant flow diagram.

Thirty participants met the eligibility criteria and were included in the study. Participants were allocated to the slider (n = 10), tensioner (n = 10), or static stretching (n = 10) intervention groups. All participants completed the intervention and post-intervention assessments, and all were included in the final analyses. A flow diagram illustrating participant recruitment, allocation, follow-up, and analysis is presented in Figure 5.

The distribution of physical activity levels according to the IPAQ is presented in Table 2. Prior to statistical analyses, the assumptions of normality (Shapiro–Wilk test and visual inspection of Q–Q plots), homogeneity of variance (Levene’s test), and homogeneity of covariance matrices (Box’s M test) were examined and considered acceptable for all continuous variables. Group differences for continuous variables were evaluated using one-way analysis of variance (ANOVA), whereas categorical variables were analyzed using appropriate contingency-table tests. Sex distribution was compared using Pearson’s chi-square test, whereas IPAQ classification was compared using the Fisher–Freeman–Halton exact test because of the small expected cell frequencies.

Overall, most participants were classified as having high physical activity levels (56.7%), followed by moderate (23.3%) and low (3.3%) physical activity levels, while 16.7% of participants did not provide sufficient information for classification. No statistically significant association between intervention group and IPAQ classification was identified using the Fisher–Freeman–Halton exact test (exact two-sided *p* = 0.714). Similarly, sex distribution was identical across intervention groups (χ^2^(2) = 0.000, *p* = 1.000). Overall, the sample included 15 male and 15 female participants, with an equal distribution within each intervention group (5 male and 5 female participants). Sex was examined descriptively as a participant characteristic; however, sex-stratified analyses of the MyotonPRO-derived outcomes were not performed because the study was not designed or powered to investigate sex-related differences. Furthermore, no significant baseline differences were identified between groups for age (F(2,27) = 0.520, *p* = 0.601), height (F(2,27) = 0.350, *p* = 0.708), or body mass (F(2,27) = 0.609, *p* = 0.551), indicating that the intervention groups were comparable at baseline. Estimated marginal means and 95% confidence intervals for all MyotonPRO-derived outcomes before and after the interventions are presented in Appendix A Table A1. Pairwise standardized differences in baseline values between intervention groups are presented in Appendix A Table A2. These standardized differences were generally small, although a moderate baseline difference was observed for rectus femoris logarithmic decrement between the tensioner and static stretching groups (Hedges’ g = −0.769).

### 3.1. Muscle Tone

**Figure 6:**
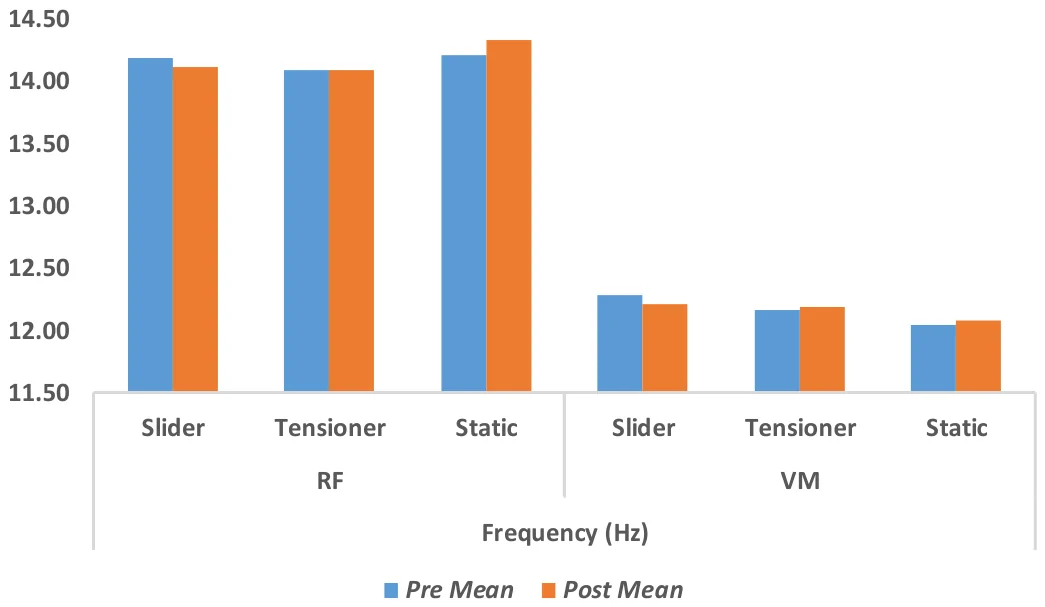
Muscle Tone (Hz) of the Rectus Femoris (RF) and Vastus Medialis (VM) before and after femoral nerve slider, femoral nerve tensioner, and static stretching interventions.

A three-way mixed repeated-measures ANOVA revealed a significant main effect of muscle on muscle tone (frequency) (F(1,27) = 129.414, *p* < 0.001, η^2^*p* = 0.827), with the rectus femoris exhibiting greater tone values than the vastus medialis. No significant main effect of time was observed (F(1,27) = 0.011, *p* = 0.918, η^2^*p* < 0.001), indicating that muscle tone did not change significantly following the interventions. Likewise, no significant main effect of intervention was detected (F(2,27) = 0.007, *p* = 0.993, η^2^*p* = 0.001). Furthermore, the time × intervention, muscle × intervention, time × muscle, and time × muscle × intervention interactions were not statistically significant (*p* > 0.05) (Figure 6).

### 3.2. Muscle Stiffness

**Figure 7:**
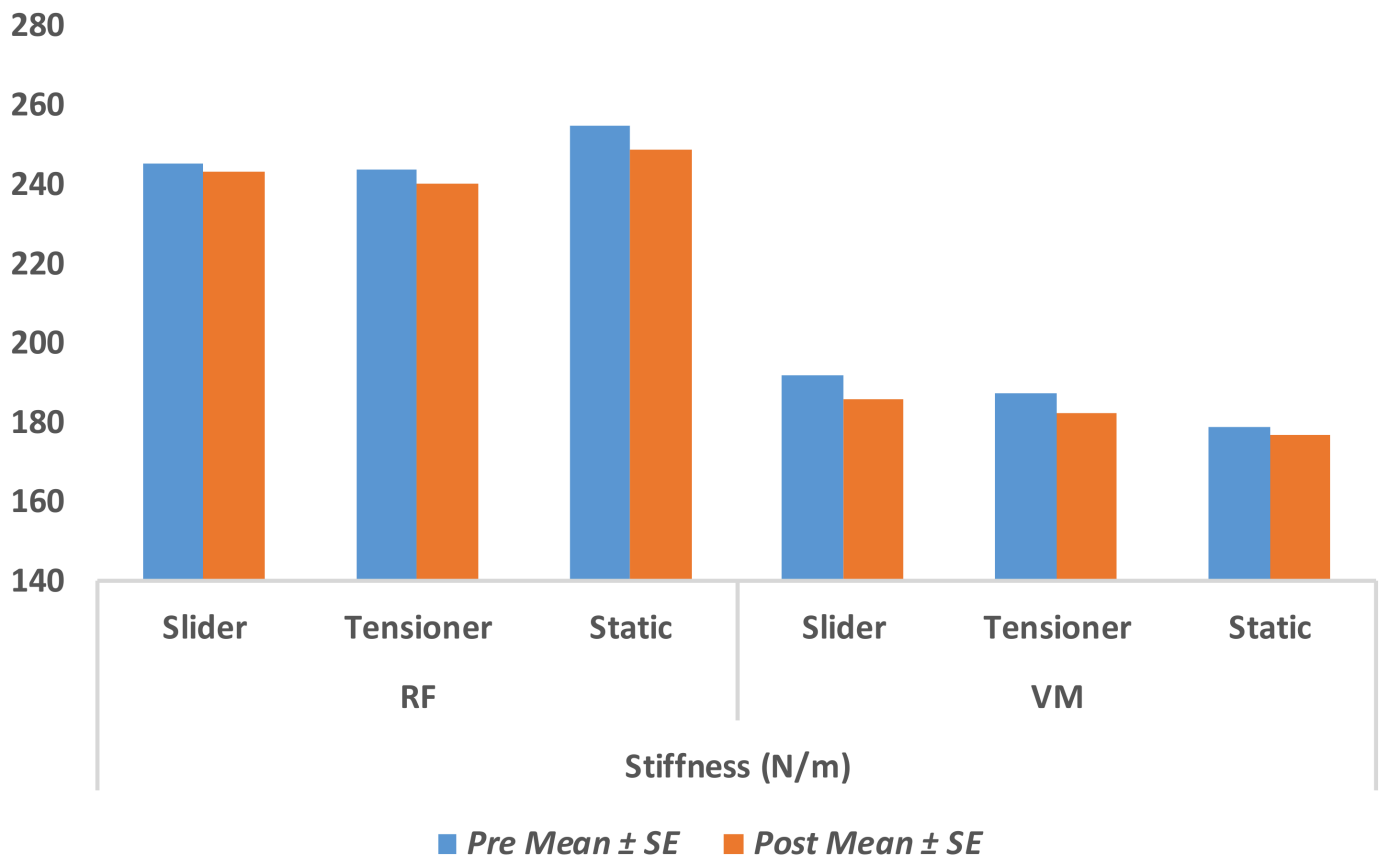
Muscle Stiffness (N/m) of the Rectus Femoris (RF) and Vastus Medialis (VM) before and after femoral nerve slider, femoral nerve tensioner, and static stretching interventions.

A significant main effect of time was observed for muscle stiffness (F(1,27) = 5.562, *p* = 0.026, η^2^*p* = 0.171), indicating an overall reduction in stiffness from pre- to post-assessment, irrespective of intervention group and muscle. A significant main effect of muscle was also detected (F(1,27) = 104.706, *p* < 0.001, η^2^*p* = 0.795), with the rectus femoris exhibiting greater stiffness than the vastus medialis. No significant main effect of intervention was found (F(2,27) = 0.016, *p* = 0.984, η^2^*p* = 0.001). Furthermore, no significant time × intervention, muscle × intervention, time × muscle, or time × muscle × intervention interactions were observed (Figure 7). Thus, although stiffness decreased overall across time, no statistically detectable intervention-specific differences in stiffness responses were observed under the conditions examined.

### 3.3. Elasticity (Logarithmic Decrement)

**Figure 8:**
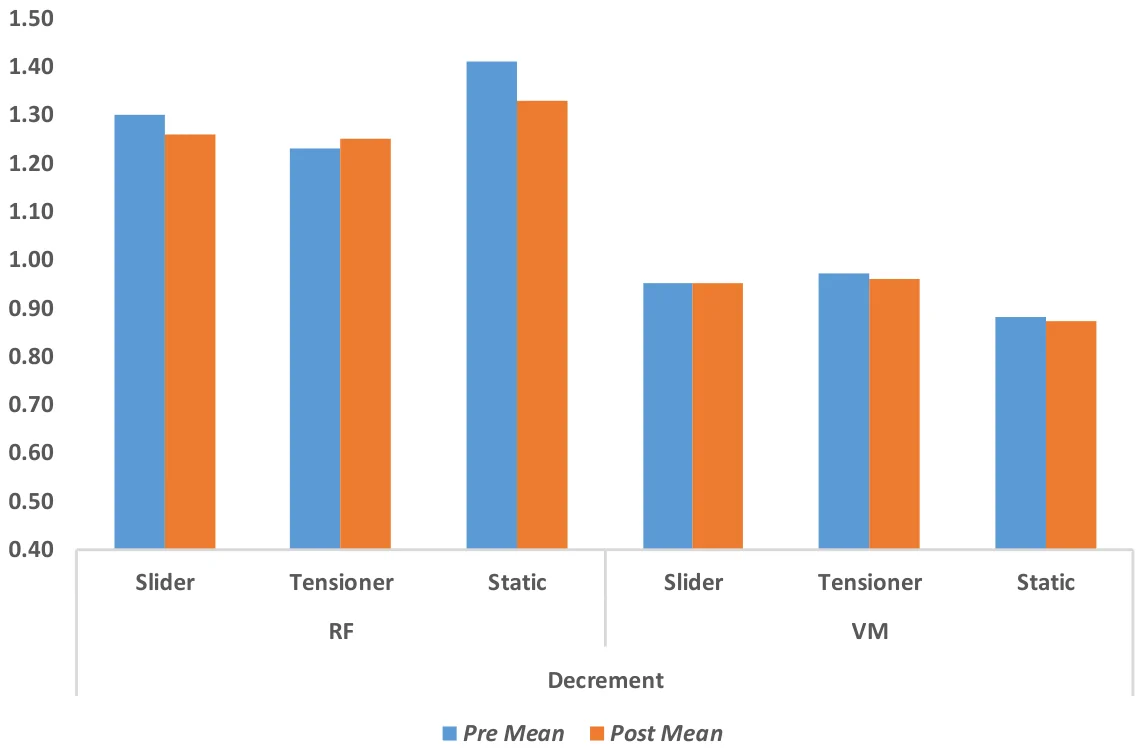
Muscle Elasticity of the Rectus Femoris (RF) and Vastus Medialis (VM) before and after femoral nerve slider, femoral nerve tensioner, and static stretching interventions.

No significant main effect of time was observed for decrement (F(1,27) = 2.629, *p* = 0.117, η^2^*p* = 0.089), indicating that decrement values did not change significantly following the interventions. A significant main effect of muscle was detected (F(1,27) = 116.771, *p* < 0.001, η^2^*p* = 0.812), with the rectus femoris demonstrating greater decrement values than the vastus medialis. No significant main effect of intervention was found (F(2,27) = 0.025, *p* = 0.975, η^2^*p* = 0.002). A significant muscle × intervention interaction was observed (F(2,27) = 3.950, *p* = 0.031, η^2^*p* = 0.226), indicating that the magnitude of the difference between muscles varied across interventions. Examination of estimated marginal means showed that the difference between the rectus femoris and vastus medialis was greatest in the static stretching group (mean difference = 0.501), compared with the slider (0.335) and tensioner (0.273) groups. No significant time × intervention interaction, time × muscle interaction, or time × muscle × intervention interaction was observed (Figure 8).

### 3.4. Relaxation Time

**Figure 9:**
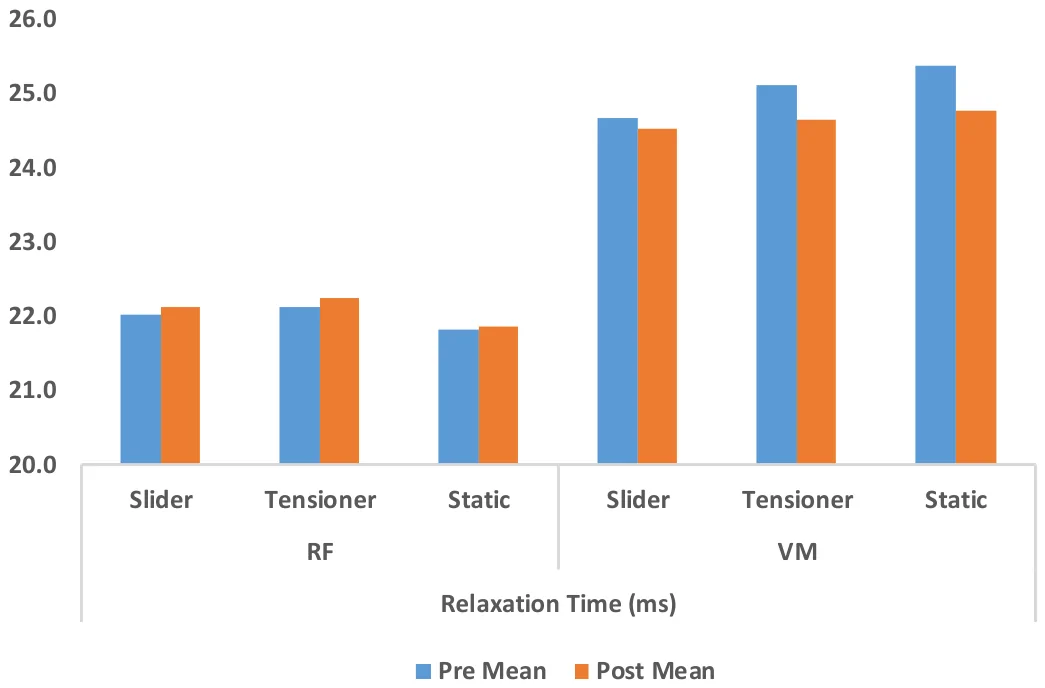
Muscle Relaxation Time (ms) of the Rectus Femoris (RF) and Vastus Medialis (VM) before and after femoral nerve slider, femoral nerve tensioner, and static stretching interventions.

No significant main effect of time was observed for relaxation time (F(1,27) = 1.595, *p* = 0.217, η^2^*p* = 0.056), indicating that relaxation time did not change significantly following the interventions. A significant main effect of muscle was detected (F(1,27) = 60.273, *p* < 0.001, η^2^*p* = 0.691), with the vastus medialis demonstrating larger relaxation time values than the rectus femoris. No significant main effect of intervention was found (F(2,27) = 0.017, *p* = 0.983, η^2^*p* = 0.001). A significant time × muscle interaction was observed (F(1,27) = 5.343, *p* = 0.029, η^2^*p* = 0.165), indicating that changes over time differed between muscles. Follow-up comparisons were adjusted for multiple testing using the Holm procedure. The pre–post reduction in relaxation time for the vastus medialis did not remain statistically significant after adjustment (mean difference = 0.40 ms, unadjusted *p* = 0.047, Holm-adjusted *p* = 0.094). Similarly, no statistically significant pre–post change was observed for the rectus femoris (mean difference = 0.09 ms, unadjusted *p* = 0.477, Holm-adjusted *p* = 0.477). No significant time × intervention interaction, muscle × intervention interaction, or time × muscle × intervention interaction was observed (Figure 9).

### 3.5. Creep

**Figure 10:**
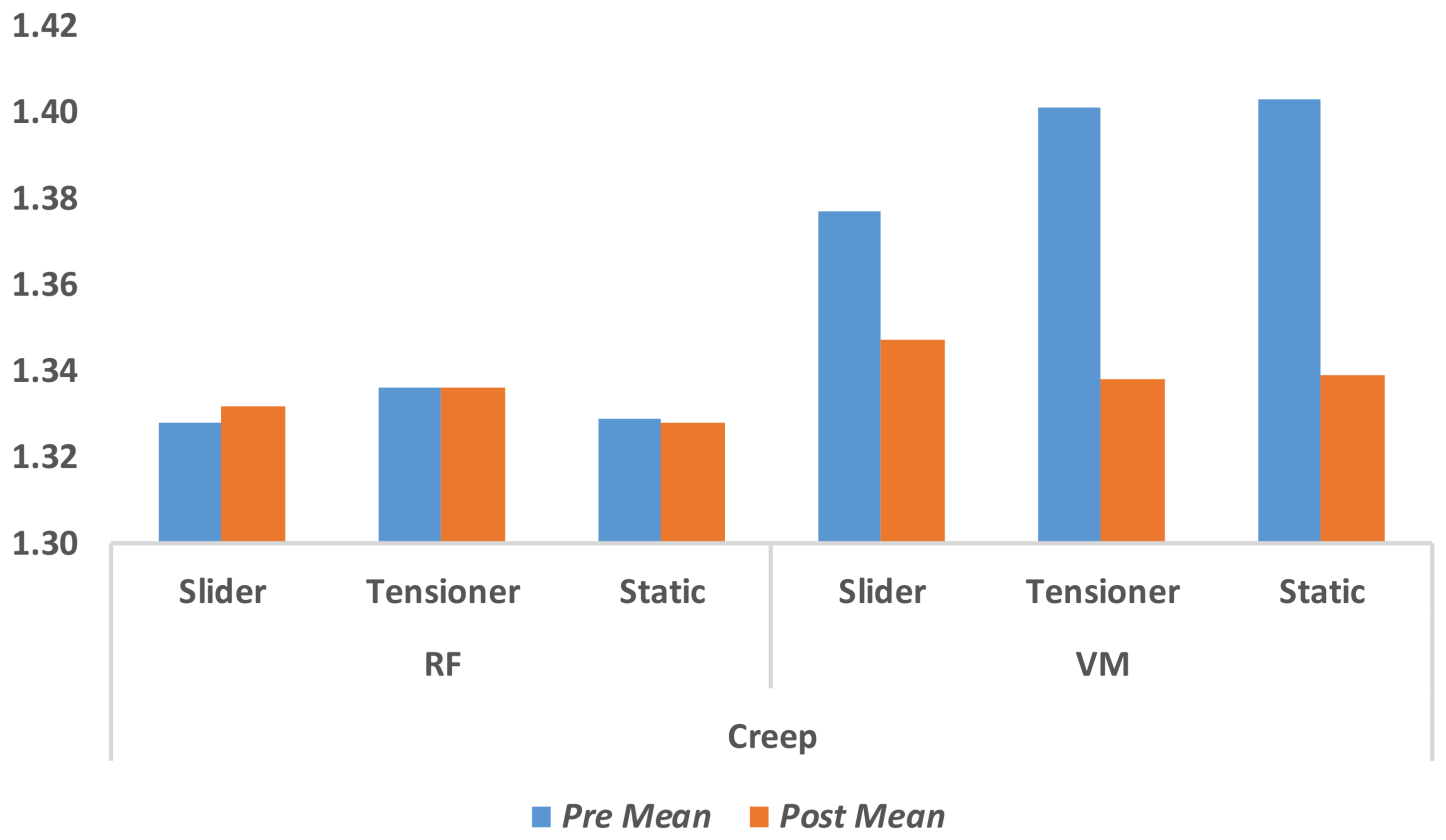
Muscle Creep of the Rectus Femoris (RF) and Vastus Medialis (VM) before and after femoral nerve slider, femoral nerve tensioner, and static stretching interventions.

A significant main effect of time was observed for creep (F(1,27) = 9.524, *p* = 0.005, η^2^*p* = 0.261), indicating an overall reduction in creep values from pre- to post-assessment. No significant main effect of muscle was detected (F(1,27) = 0.992, *p* = 0.328, η^2^*p* = 0.035), and no significant time × intervention interaction was found (F(2,27) = 0.581, *p* = 0.566, η^2^*p* = 0.041). A significant time × muscle interaction was observed (F(1,27) = 11.574, *p* = 0.002, η^2^*p* = 0.300), indicating that changes over time differed between muscles. Follow-up comparisons were adjusted for multiple testing using the Holm procedure. No statistically significant pre–post change was observed for the rectus femoris (unadjusted *p* = 0.867, Holm-adjusted *p* = 0.867), whereas the vastus medialis demonstrated a significant reduction in creep from pre- to post-assessment (mean difference = 0.052, unadjusted *p* = 0.002, Holm-adjusted *p* = 0.004). No significant muscle × intervention interaction or time × muscle × intervention interaction was observed (Figure 10).

The estimated marginal means (±SE) for frequency, stiffness, decrement, relaxation time, and creep in the rectus femoris and vastus medialis muscles before and after the interventions are shown in Appendix A. Across all outcomes, pre- and post-intervention values were generally similar among the three intervention groups. Consistent with the mixed ANOVA results, no significant time × intervention interactions were observed for any variable, indicating that the magnitude of change over time did not differ between the slider, tensioner, and static stretching interventions.

## 4. Discussion

Although neurodynamic techniques and static stretching are widely used to modify lower-limb mobility and flexibility, their immediate effects on the mechanical properties of skeletal muscle remain insufficiently understood. Previous research has primarily focused on clinical or functional outcomes, such as range of motion and flexibility, while comparatively little is known about whether neurodynamic techniques produce measurable alterations in muscle tone, stiffness, elasticity, and viscoelastic behavior, or whether these responses differ from those elicited by conventional static stretching. Clarifying these responses is relevant because objective characterization of the immediate mechanical effects of these interventions may improve understanding of their neuromechanical consequences and provide a basis for future research examining their potential clinical and functional relevance.

The present study investigated the acute effects of femoral nerve slider, femoral nerve tensioner, and static stretching interventions on the mechanical properties of the rectus femoris and vastus medialis muscles using MyotonPRO assessment. Contrary to the initial hypothesis, no statistically detectable intervention-specific effects were identified, as no significant time × intervention or time × muscle × intervention interactions were observed for any of the examined mechanical parameters. Significant temporal changes were identified for selected outcomes, including stiffness and creep, together with time × muscle interactions for relaxation time and creep. However, because the study did not include a sham or no-intervention control condition, these temporal effects cannot be attributed specifically to the interventions. Repeated measurement, participant relaxation, familiarization with the testing procedure, maintenance of the testing position, or the passage of time may also have contributed to the observed changes. Accordingly, the findings should primarily be interpreted as demonstrating an absence of statistically detectable differences in the immediate mechanical responses among the three intervention procedures.

A significant reduction in muscle stiffness was observed over time, irrespective of intervention group. However, no significant time × intervention or other interaction involving intervention was detected; thus, no statistically detectable intervention-specific differences in stiffness responses were observed under the conditions examined. Muscle stiffness reflects resistance to external deformation and is considered an important indicator of passive mechanical behavior and neuromuscular readiness [34]. The observed reduction therefore represents an acute temporal change in measured passive mechanical behavior; however, its physiological and clinical significance remains uncertain [35].

The present findings are partially consistent with those reported by Satkunskiene, Khair, Muanjai, Mickevicius and Kamandulis [35], who compared neurodynamic nerve gliding with static stretching and demonstrated that both interventions could influence tissue mechanical behavior, although intervention-specific differences were identified for certain neuromechanical outcomes. In contrast, the present study did not identify statistically detectable intervention-specific differences in stiffness responses under the conditions examined.

The rectus femoris consistently demonstrated greater stiffness values than the vastus medialis. These differences may relate to the distinct anatomical and architectural characteristics of the two muscles, including the biarticular function of the rectus femoris and differences in muscle architecture [36,37,38]. However, muscle architecture and tissue composition were not directly assessed in the present study; therefore, these potential explanations remain speculative.

Logarithmic decrement did not demonstrate a significant temporal change, indicating no statistically detectable pre–post alteration in this parameter. The rectus femoris demonstrated larger decrement values than the vastus medialis, reflecting differences in the measured elasticity-related parameter between the two muscles. Although a significant muscle × intervention interaction was observed, this was not accompanied by a significant time × muscle × intervention interaction and therefore does not indicate differential intervention-induced changes over time.

A significant time × muscle interaction was observed for relaxation time, indicating that the temporal response differed between the rectus femoris and vastus medialis. However, after adjustment for multiple comparisons using the Holm procedure, neither the pre–post change in the vastus medialis nor that in the rectus femoris remained statistically significant. Therefore, although the significant interaction suggests a differential temporal pattern between muscles, the individual muscle-specific changes should be interpreted cautiously. Relaxation time reflects the ability of tissue to recover following deformation and provides information regarding tissue viscoelastic behaviour [39,40]. However, the physiological and clinical significance of the observed reduction in relaxation time remains uncertain. Importantly, this muscle-specific temporal pattern was not dependent on intervention type, as no significant time × intervention or time × muscle × intervention interaction was observed. Therefore, rather than indicating that the vastus medialis is inherently more responsive to acute mobilization, these findings indicate that the temporal response differed between the two muscles under the conditions examined. Anatomical and functional differences between the rectus femoris and vastus medialis, including variations in muscle architecture, tissue composition, and loading characteristics, may provide a plausible explanation for such muscle-specific temporal patterns [41]; however, these mechanisms were not directly examined in the present study and should therefore be considered hypothesis-generating.

An additional finding of the present study concerned creep behavior. A significant main effect of time and a significant time × muscle interaction were observed. Follow-up analyses adjusted using the Holm procedure demonstrated that creep decreased significantly from pre- to post-assessment in the vastus medialis (adjusted *p* = 0.004), whereas no significant temporal change was observed in the rectus femoris. Importantly, no intervention-specific effects were identified. Creep is a viscoelastic parameter derived from the Deborah number and reflects the deformation and recovery behavior of biological tissues following a brief mechanical impulse [40,42]. Although the physiological interpretation of MyotonPRO-derived creep remains incompletely understood, it may provide information regarding tissue viscoelastic behaviour [39,40].

The reduction in creep observed in the vastus medialis indicates an acute pre–post change in the measured viscoelastic parameter; however, the physiological and clinical significance of this change remains uncertain. Because no sham or no-intervention control group was included, this temporal change cannot be attributed specifically to the interventions and may, at least partly, reflect non-specific influences such as repeated measurement, participant relaxation, the passage of time, or measurement variability. The vastus medialis demonstrated a significant pre–post reduction in creep that remained significant after correction for multiple comparisons, whereas the corresponding pre–post change in relaxation time did not remain statistically significant after Holm correction. Nevertheless, significant time × muscle interactions were observed for both parameters, indicating that their temporal patterns differed between the rectus femoris and vastus medialis. Importantly, these muscle-specific temporal patterns were not dependent on intervention type, as no significant time × muscle × intervention interactions were observed. Therefore, rather than suggesting that the vastus medialis is inherently more responsive to acute mechanical or neurodynamic stimuli, these findings indicate that muscle-specific temporal responses may differ across mechanical parameters under the conditions examined. The mechanisms underlying these differences and their physiological or clinical relevance require further investigation.

The absence of significant intervention-specific effects across all examined variables is an important finding. A growing body of literature supports the effectiveness of neurodynamic techniques for improving flexibility and range of motion. Ahmed and Samhan [43] reported that both neurodynamic stretching and static stretching significantly improved hamstring flexibility, with neurodynamic stretching producing superior gains. More recently, Bertacchini et al. [44], in a systematic review and meta-analysis including 30 randomized controlled trials and 1.379 participants, concluded that neurodynamic interventions were superior to static stretching for improving hamstring flexibility, with slider techniques demonstrating marginally greater effectiveness than tensioners. However, the outcomes assessed in these studies primarily reflected flexibility and range-of-motion measures rather than objective muscle mechanical properties. Consequently, the present findings suggest that improvements in flexibility do not necessarily translate into differential changes in muscle mechanical behavior.

Although neurodynamic techniques may increase neural excursion, reduce mechanosensitivity, and improve range of motion, these mechanisms may not be sufficient to induce distinct acute changes in muscle tone, stiffness, elasticity, relaxation time, or creep as assessed by MyotonPRO. This interpretation is supported by previous work demonstrating that flexibility is influenced not only by the viscoelastic characteristics of muscle and connective tissue but also by stretch tolerance and the interaction between muscles and adjacent neural structures [45]. Furthermore, Satkunskiene, Khair, Muanjai, Mickevicius and Kamandulis [35] reported that both neurodynamic mobilization and static stretching influenced neuromechanical properties, although only limited intervention-specific differences were observed.

Static stretching and neurodynamic techniques are proposed to influence mobility through different mechanisms. Static stretching has traditionally been associated with changes in tissue extensibility and viscoelastic behaviour [45], whereas neurodynamic techniques are proposed to influence neural excursion and mechanosensitivity [1,44,46,47]. Moreover, slider and tensioner techniques are theoretically differentiated by their effects on neural excursion and strain [3,5]. However, these mechanisms were not directly assessed in the present study. Therefore, the absence of statistically detectable intervention-specific differences in MyotonPRO-derived outcomes should not be interpreted as evidence that these interventions act through similar physiological mechanisms. Rather, the present findings are restricted to the immediate muscle mechanical properties assessed under the conditions examined.

From a clinical perspective, neurodynamic interventions may still offer practical advantages despite producing similar acute changes in muscle mechanical properties to static stretching. Several systematic reviews have demonstrated that prolonged static stretching can acutely reduce maximal force production, power output, sprint performance, and explosive athletic performance, particularly when stretches are held for longer durations [48,49,50]. In contrast, current evidence suggests that neurodynamic techniques can improve range of motion and flexibility without the performance decrements commonly associated with static stretching [28,51,52]. Consequently, neurodynamic interventions may represent an attractive alternative when the objective is to enhance mobility while minimizing potential negative effects on subsequent strength and athletic performance. Nevertheless, this hypothesis remains speculative because the present study did not evaluate strength, power, or functional performance outcomes. Future studies should directly compare the effects of neurodynamic techniques and static stretching on both muscle mechanical properties and performance-related variables to determine whether neurodynamic interventions provide functional advantages beyond those identified through MyotonPRO assessments.

### 4.1. Clinical Implications

The present findings indicated that no statistically detectable intervention-specific differences in acute quadriceps mechanical responses were observed among femoral nerve slider techniques, tensioner techniques, and static stretching in healthy individuals. From a clinical perspective, this suggests that when the immediate modification of quadriceps mechanical properties is the primary consideration, the present findings do not provide evidence to favor one of these approaches over another. Consequently, selection of an intervention may need to be guided by other clinically relevant factors, such as the therapeutic objective, individual tolerance, symptom response, and the specific functional outcome being targeted. However, these findings should not be interpreted as evidence of equivalence among the interventions. Moreover, because the study involved healthy individuals and did not assess pain, range of motion, neural mechanosensitivity, strength, or functional performance, the clinical relevance of the observed mechanical responses remains uncertain. Rather, the findings provided preliminary information regarding the immediate mechanical responses of healthy quadriceps muscle under the tested conditions. Future studies incorporating symptomatic populations and clinically relevant outcomes are required to determine whether these interventions differ in their therapeutic effects.

### 4.2. Limitations

Several limitations should be acknowledged. First, knee flexion range of motion was not assessed. Although knee flexion range of motion was prospectively registered as a secondary outcome, this measure was not collected because priority was given to completing the MyotonPRO reassessment within the short post-intervention window. This protocol deviation limited the ability to determine whether the observed mechanical responses were accompanied by changes in joint mobility. Second, only the immediate effects of the interventions were examined; therefore, the persistence of the observed responses remains unknown.

Third, MyotonPRO measurements provide indirect estimates of tissue mechanical properties and do not allow for direct assessment of neural excursion or neural mechanosensitivity. In addition, subcutaneous adipose tissue thickness at the measurement sites was not quantified. Because the MyotonPRO assesses mechanical responses through the overlying soft tissues, potential variability associated with differences in subcutaneous tissue thickness cannot be excluded. Additionally, assessor blinding was not feasible because the same investigator administered the interventions and performed the MyotonPRO assessments. Although standardized measurement procedures and consistent anatomical measurement sites were used to minimize measurement variability, the possibility of assessor-related measurement bias cannot be completely excluded. Furthermore, participants were assigned using a predetermined alternating sequence rather than random allocation, and the absence of allocation concealment may have introduced selection bias that should be considered when interpreting the internal validity of the findings. Although standardized baseline differences were generally small, a moderate between-group difference was observed for rectus femoris logarithmic decrement, indicating that some baseline imbalance cannot be excluded. In addition, although the intervention procedures were standardized with respect to participant positioning, movement sequence, cadence, and duration, movement amplitude and perceived stretching intensity were not objectively quantified. Consequently, identical mechanical loading or intervention intensity across participants cannot be assumed.

Fourth, the study was conducted in healthy participants; therefore, the findings should not be extrapolated directly to symptomatic populations. Fifth, although the a priori sample size calculation indicated that 30 participants were sufficient under the prespecified assumptions, sensitivity analysis based on the achieved sample indicated that the study had 80% power to detect Time × Intervention effects of f = 0.300 or greater. Therefore, smaller intervention-specific and higher-order interaction effects may have gone undetected, and the absence of statistically significant interaction effects should not be interpreted as evidence of equivalence among the interventions.

Finally, an important limitation is the absence of a sham or no-intervention control group. Consequently, it cannot be determined whether the observed temporal changes in muscle mechanical properties were attributable to the interventions or reflected, at least partly, non-specific effects associated with repeated measurements, participant relaxation, familiarization, maintenance of the testing position, or the passage of time. However, the primary aim of the study was to compare the acute effects of different intervention modalities rather than to establish their efficacy relative to no treatment. Therefore, the findings should be interpreted as indicating that no statistically significant differences in pre–post changes were detected among the femoral nerve slider, femoral nerve tensioner, and static stretching groups under the conditions examined, rather than as evidence of equivalence between the interventions.

### 4.3. Future Directions

Future studies should investigate the long-term effects of femoral nerve neurodynamic interventions, include symptomatic populations, and combine mechanical assessments with measures of range of motion, neural mobility, ultrasound elastography, electromyography, and clinical outcomes. In addition to cross-sectional comparisons, longitudinal and repeated-measures studies examining intra-subject changes should be conducted to determine how muscle mechanical properties change within the same individuals following single and repeated intervention sessions. Such designs may also help characterize the magnitude, time course, and persistence of individual responses to neurodynamic and stretching interventions. Such approaches may provide greater insight into the mechanisms underlying neurodynamic treatment effects and clarify whether changes in muscle mechanical properties are associated with meaningful functional improvements.

## 5. Conclusions

The present study found no statistically detectable intervention-specific differences in the immediate MyotonPRO-derived mechanical properties of the rectus femoris and vastus medialis following femoral nerve slider, femoral nerve tensioner, or static stretching procedures in healthy young adults. Significant temporal changes were observed for stiffness and creep, while muscle-specific temporal responses were identified for relaxation time and creep. However, in the absence of a sham or no-intervention control condition, these temporal changes cannot be attributed specifically to the interventions and may partly reflect non-specific influences associated with repeated measurement, participant relaxation, or the passage of time. Furthermore, the absence of significant intervention-specific interactions should not be interpreted as evidence of equivalence among the three procedures. The observed differences between the rectus femoris and vastus medialis suggest that muscle-specific mechanical characteristics may warrant further investigation; however, their physiological and clinical significance remains uncertain. As the present study included healthy participants and did not assess pain, range of motion, neural mechanosensitivity, strength, or functional performance, the findings should not be extrapolated directly to symptomatic populations or clinical effectiveness. Future studies should investigate longer-term responses, include sham or no-intervention conditions and symptomatic populations, and examine whether changes in muscle mechanical properties are associated with clinically and functionally meaningful outcomes.

## Figures and Tables

**Table 1:** Mechanical properties quantified by the MyotonPRO device and their physiological interpretation.

Parameter (Unit)	What It Measures	Physiological Interpretation
Frequency (Hz)	Natural oscillation frequency of the tissue at rest	Represents the intrinsic muscle tone or passive tension of the muscle without voluntary contraction
Dynamic Stiffness (N/m)	Resistance of the tissue to an external mechanical force	Reflects the mechanical resistance of the muscle to deformation
Logarithmic Decrement (Arbitrary units)	Damping of tissue oscillation after mechanical impulse	Represents energy dissipation during oscillation and is inversely related to elastic behaviour
Relaxation Time (ms)	Time required for tissue to recover after deformation	Reflects viscoelastic recovery following mechanical loading
Creep (Arbitrary units)	Ratio of mechanical stress relaxation time to deformation time (Deborah number)	Represents a MyotonPRO-derived index of tissue viscoelastic behaviour following a brief mechanical impulse

Abbreviations: Hz, hertz; N/m, newtons per meter; ms, milliseconds.

**Table 2:** Characteristics of the included sample.

Variable	Slider (n = 10)	Tensioner (n = 10)	Static Stretching (n = 10)	*p*-Value
Age (years), mean ± SD	21.20 ± 0.42	21.70 ± 0.95	21.20 ± 1.93	0.601
Sex (Male/Female), n	5/5	5/5	5/5	1.000
Height (cm), mean ± SD	170.0 ± 10.1	170.7 ± 8.8	173.3 ± 8.9	0.708
Body Mass (kg), mean ± SD	68.0 ± 14.9	67.7 ± 15.3	73.7 ± 10.4	0.551
IPAQ Classification, n (%)				
High	7 (70%)	5 (50%)	5 (50%)	0.714 ^a^
Moderate	1 (10%)	3 (30%)	3 (30%)	
Low	0 (0%)	1 (10%)	0 (0%)	
Not reported	2 (20%)	1 (10%)	2 (20%)	

Abbreviations: IPAQ, International Physical Activity Questionnaire; SD, standard deviation; n, number. ^a^ Fisher–Freeman–Halton exact test.

## Data Availability

The data presented in this study are available upon request from the corresponding author.

## Supplementary Materials

The following supporting information can be downloaded at: https://www.mdpi.com/article/10.3390/bioengineering13080927/s1.

## Abbreviations

The following abbreviations are used in this manuscript:

ANOVA	Analysis of Variance
bpm	Beats Per Minute
IPAQ-SF	International Physical Activity Questionnaire—Short Form
Hz	Hertz
N/m	Newtons per Meter
ms	Milliseconds
RF	Rectus Femoris
VM	Vastus Medialis
SD	Standard Deviation
η^2^*p*	Partial Eta Squared
Q–Q	Quantile–Quantile
MyotonPRO	MyotonPRO Handheld Myotonometer
TCTR	Thai Clinical Trials Registry
SPSS	Statistical Package for the Social Sciences
ICC	Intraclass Correlation Coefficient

## Appendix A

**Table A1:** Estimated marginal means and 95% confidence intervals for muscle mechanical properties before and after the interventions.

Variable	Muscle	Intervention	Pre EMM (95% CI)	Post EMM (95% CI)	Descriptive Mean Change
**Frequency (Hz)**	RF	Slider	14.18 (13.10–15.26)	14.11 (12.97–15.25)	↓ 0.07
		Tensioner	14.08 (13.00–15.16)	14.08 (12.94–15.22)	No change
		Static	14.21 (13.13–15.29)	14.32 (13.18–15.46)	↑ 0.11
	VM	Slider	12.28 (11.64–12.92)	12.21 (11.48–12.94)	↓ 0.07
		Tensioner	12.16 (11.52–12.80)	12.18 (11.45–12.91)	↑ 0.02
		Static	12.04 (11.40–12.68)	12.08 (11.35–12.81)	↑ 0.04
**Stiffness (N/m)**	RF	Slider	245.20 (221.19–269.21)	243.10 (217.25–268.96)	↓ 2.10
		Tensioner	243.50 (219.49–267.51)	240.20 (214.35–266.06)	↓ 3.30
		Static	254.60 (230.59–278.61)	248.70 (222.85–274.56)	↓ 5.90
	VM	Slider	191.60 (163.25–219.95)	185.70 (155.27–216.13)	↓ 5.90
		Tensioner	187.30 (158.95–215.65)	182.30 (151.87–212.73)	↓ 5.00
		Static	178.60 (150.25–206.95)	176.50 (146.07–206.93)	↓ 2.10
**Decrement**	RF	Slider	1.307 (1.170–1.444)	1.268 (1.127–1.409)	↓ 0.039
		Tensioner	1.237 (1.100–1.374)	1.252 (1.111–1.393)	↑ 0.015
		Static	1.418 (1.281–1.555)	1.338 (1.197–1.479)	↓ 0.080
	VM	Slider	0.951 (0.797–1.105)	0.953 (0.806–1.100)	↑ 0.002
		Tensioner	0.976 (0.822–1.130)	0.967 (0.820–1.114)	↓ 0.009
		Static	0.883 (0.729–1.037)	0.871 (0.724–1.018)	↓ 0.012
**Relaxation Time (ms)**	RF	Slider	22.02 (20.24–23.81)	22.12 (20.20–24.04)	↑ 0.100
		Tensioner	22.11 (20.33–23.90)	22.23 (20.31–24.15)	↑ 0.120
		Static	21.81 (20.03–23.60)	21.86 (19.94–23.78)	↑ 0.050
	VM	Slider	24.66 (23.09–26.23)	24.52 (23.05–26.00)	↓ 0.140
		Tensioner	25.11 (23.54–26.68)	24.64 (23.17–26.12)	↓ 0.470
		Static	25.36 (23.79–26.93)	24.77 (23.30–26.25)	↓ 0.590
**Creep**	RF	Slider	1.328 (1.234–1.422)	1.332 (1.234–1.430)	↑ 0.004
		Tensioner	1.336 (1.242–1.430)	1.336 (1.238–1.434)	No change
		Static	1.329 (1.235–1.423)	1.328 (1.230–1.426)	↓ 0.001
	VM	Slider	1.377 (1.268–1.486)	1.347 (1.246–1.448)	↓ 0.030
		Tensioner	1.401 (1.292–1.510)	1.338 (1.237–1.439)	↓ 0.063
		Static	1.403 (1.294–1.512)	1.339 (1.238–1.440)	↓ 0.064

Values are presented as estimated marginal means (EMMs) with 95% confidence intervals (CIs). Mean change was calculated as post-assessment minus pre-assessment. Mean changes for individual intervention groups are descriptive and should not be interpreted as statistically significant within-group effects. RF, rectus femoris; VM, vastus medialis.

**Table A2:** Baseline standardized differences.

Variable	Muscle	Slider vs. Tensioner	Slider vs. Static	Tensioner vs. Static
Frequency (Hz)	RF	0.057	−0.017	−0.079
	VM	0.112	0.227	0.124
Stiffness (N/m)	RF	0.048	−0.226	−0.287
	VM	0.091	0.275	0.207
Decrement	RF	0.366	−0.482	−0.769
	VM	−0.100	0.291	0.358
Relaxation time (ms)	RF	−0.033	0.070	0.103
	VM	−0.177	−0.256	−0.110
Creep	RF	−0.057	−0.006	0.045
	VM	−0.137	−0.139	−0.012

Values are Hedges’ g. Positive values indicate higher baseline values in the first-listed group and negative values higher baseline values in the second-listed group.
